# Pancreatic amylin dynamically reconfigures distributed brain networks governing appetite regulation in mice

**DOI:** 10.1016/j.molmet.2025.102313

**Published:** 2025-12-22

**Authors:** Irmak Gezginer, Giulia Mazzini, Christelle Le Foll, Diana Kindler, Thomas A. Lutz, Daniel Razansky

**Affiliations:** 1 Institute for Biomedical Engineering and Institute of Pharmacology and Toxicology, Faculty of Medicine, University of Zurich, Switzerland; 2 Institute for Biomedical Engineering, Department of Information Technology and Electrical Engineering, ETH Zurich, Switzerland; 3 Institute of Veterinary Physiology, Vetsuisse Faculty, University of Zurich, Zurich, Switzerland

**Keywords:** Obesity, Amylin, Functional magnetic resonance imaging, Functional connectivity, Receptor activity-modifying protein

## Abstract

Obesity remains a major global health challenge, yet the brain-wide effects of hormones regulating appetite remain incompletely understood. Amylin, co-secreted with insulin by pancreatic β-cells, promotes satiation and is a promising therapeutic target for metabolic disorders. While its receptor distribution is well-characterized, its influence on large-scale neural dynamics is unknown. Here, resting-state fMRI was used to map time-resolved connectivity changes following peripheral amylin administration in wild-type (WT) and receptor activity-modifying protein 1/3 knockout (RAMP1/3 KO) mice. In WT animals, amylin triggered rapid and transient network reconfigurations, engaging canonical satiation hubs such as the area postrema and parabrachial nucleus, and extending to sensory-integrative areas including the inferior colliculus and insular cortex. Early hindbrain responses propagated to hypothalamic, thalamic, and mesolimbic circuits implicated in appetite and reward. These effects, along with amylin-driven modulation of large-scale networks and low-frequency oscillations, were absent in KO mice. The findings position amylin as a potent modulator of distributed brain circuits, offering a framework for targeted obesity treatments.

## Highlights

• Peripheral amylin induces rapid, transient reconfiguration of brain-wide networks in mice.
• Connectivity shifts extend from hindbrain entry points to hypothalamic and reward hubs.
• Amylin effects are absent in RAMP1/3 KO mice, confirming receptor dependence.
• Amylin enhances slow brain oscillations linked to satiation and appetite control.

## 1 Introduction

Obesity remains one of the most pressing global health challenges, contributing significantly to type 2 diabetes, cardiovascular disease, and certain cancers [1]. Because food intake is critical for maintaining energy balance, the brain must continuously integrate signals indicating the body's metabolic state to coordinate feeding behavior [2]. Thus, effective weight-control strategies depend on a mechanistic understanding of the neural circuits governing appetite, satiation and satiety.

A range of peripherally secreted hormones, including leptin, insulin, ghrelin, peptide YY, and glucagon-like peptide 1 (GLP-1), regulate energy homeostasis by conveying metabolic information to the central nervous system [[3], [4], [5], [6]]. These signals converge on brain regions such as the arcuate nucleus of the hypothalamus (ARC), area postrema (AP), and nucleus of the solitary tract (NTS), which play key roles in satiation regulation [[7], [8], [9]]. In parallel, dopaminergic circuits in the ventral tegmental area (VTA) and nucleus accumbens (NAc) modulate the hedonic and motivational aspects of feeding, influencing food-seeking behavior and reward-driven intake [[10], [11], [12]]. These homeostatic and hedonic systems exhibit substantial crosstalk, with metabolic hormones modulating dopaminergic tone and vice versa [[13], [14], [15]].

Among these peripheral signals, amylin (also called islet amyloid polypeptide, or IAPP) has emerged as a critical regulator of feeding behavior. Co-secreted with insulin from pancreatic β-cells in response to nutrient intake, amylin acts rapidly, with a half-life of approximately 13 min in rodents [16,17]. In both rodents and humans, peripheral administration of amylin reduces food intake, delays gastric emptying, and suppresses postprandial glucagon secretion, underscoring its potential in managing metabolic disorders such as type 2 diabetes [18,19]. Growing evidence also suggests long-term effects on body weight, positioning amylin analogs as promising pharmacotherapies for obesity [[20], [21], [22]].

**Figure 1:**
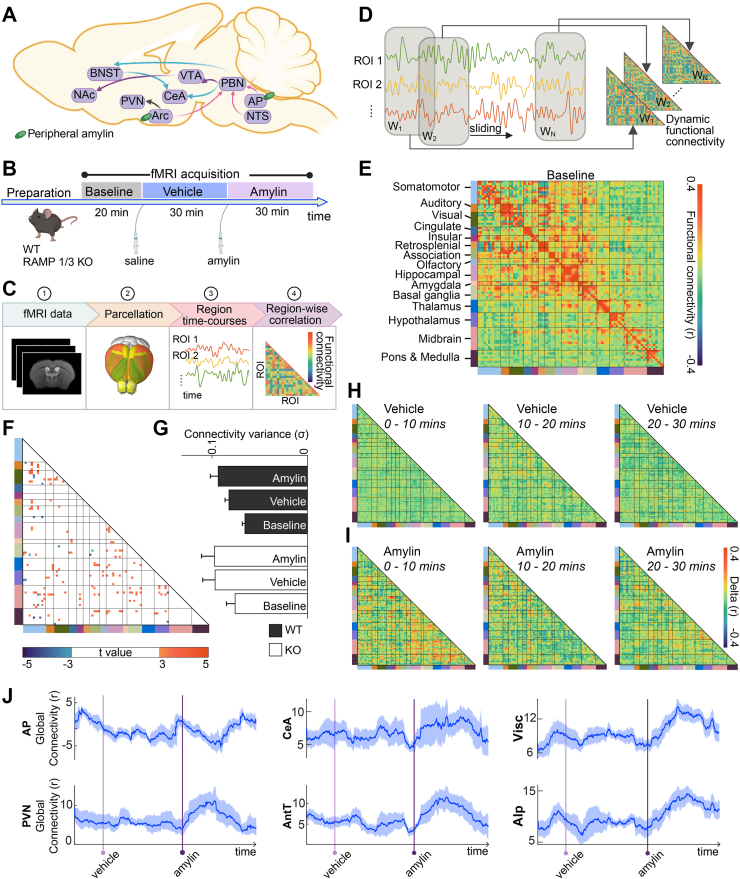
**Whole-brain neural correlates of peripheral amylin administration by means of functional connectivity. A** Amylin brain circuitry (adapted from [28]). Peripheral amylin engages a distributed network of regions involved in feeding regulation. The area postrema (AP) and nucleus of the solitary tract (NTS) act as primary entry points, relaying signals to the parabrachial nucleus (PBN). Downstream projections extend to the central amygdala (CeA) and bed nucleus of the stria terminalis (BNST), while parallel modulation of reward-related circuits involves the ventral tegmental area (VTA) and nucleus accumbens (NAc). Amylin also acts on the arcuate nucleus (ARC), influencing the paraventricular hypothalamic nucleus (PVN) to promote satiation and energy expenditure. **B** Experimental paradigm. Wild-type (WT; n = 7) and RAMP1/3 knockout (KO; n = 7) mice underwent serial fMRI scanning: 20-minute baseline, followed by vehicle injection and 30-minute acquisition, then amylin injection and a final 30-minute scan. **C** Whole-brain fMRI data were parcellated into anatomically defined regions. Functional connectivity (FC) was computed using Pearson's correlation between regional time series. **D** Dynamic FC was assessed via a sliding-window approach, generating time-resolved connectivity matrices that capture FC fluctuations across the experiment. **E** Baseline FC matrix in WT mice. Network organization is color-coded. **F** FC difference map showing baseline contrast between KO and WT animals. **G** FC variability (mean ± SEM) across conditions in WT and KO mice. Both groups showed increased variance after vehicle injection, but only WT mice exhibited further variability after amylin. **H-I** Temporal modulation of FC in WT mice following vehicle (H) and amylin (I) administration. FC was computed in consecutive 10-minute segments and compared to the preceding interval. Amylin induced widespread FC shifts during the initial 10-minute, partially reversing in the subsequent 10–20 minute period. **J** Global FC changes in WT animals across regions (mean ± SEM). Global FC reflects whole-brain coupling strength, calculated by summing each ROI's Fisher z-transformed connectivity with all other ROIs. Following amylin administration, temporary alterations in overall connectivity was observed in the AP, CeA, visceral cortex (Visc), PVN, anterior dorsal thalamus (AntT), and posterior agranular insula (AIp).

Amylin exerts its effects via a multi-subunit heterodimer receptor complex composed of the calcitonin receptor (CTR) and one of three receptor activity-modifying proteins (RAMP1–3), forming pharmacologically distinct subtypes (AMY_1-3_R) [23,24]. Mice lacking both RAMP1 and RAMP3 (RAMP1/3 double-knockout, KO) have been instrumental in mapping central amylin signaling, while RAMP2 deletion is embryonically lethal. In these models, peripheral amylin elicits reduced c-Fos activation compared to wild-type (WT) animals [25]. Recently, we have shown that cagrilintide, which in clinical trials is the most advanced amylin analogue for the treatment of obesity, also critically depends on the AMY_1/3_ receptors for its weight loss effect [26]. Prior studies have shown that peripheral amylin engages a distributed set of brain circuits involved in appetite, metabolism, and reward-driven feeding (Fig. 1A) [[27], [28], [29]]. The AP, a circumventricular organ outside the blood–brain barrier, represents a primary target for circulating amylin [30]. Lesion studies confirm the essential role of the AP and NTS in mediating amylin-induced anorexia [31]. From these early relay centers, amylin signals propagate to the parabrachial nucleus (PBN), central amygdala (CeA), and bed nucleus of the stria terminalis (BNST), eventually reaching reward-related areas such as the VTA and NAc [28,32]. Amylin also acts directly on hypothalamic regions like the ARC, where it suppresses orexigenic agouti-related peptide (AgRP) neurons and synergizes with leptin to reduce feeding [[33], [34], [35]].

Despite substantial progress in identifying amylin-responsive regions, little is known about how amylin modulates large-scale brain network dynamics in a time-resolved manner. Traditional techniques such as pharmacological interventions, lesion models, and immunohistochemistry provide only static snapshots of neural activity and may overlook transient but critical phases of circuit engagement [24]. In contrast, functional connectivity (FC) analysis of functional magnetic resonance imaging (fMRI) data offers a powerful framework to capture continuous changes in brain-wide activity [36,37]. By tracking the spatiotemporal evolution of blood oxygenation level–dependent (BOLD) signals after peripheral amylin injection, it becomes possible to map how brain regions are dynamically recruited or disengaged across time.

In this study, we hypothesized that peripheral amylin administration triggers a reorganization among key brain regions involved in feeding regulation. To test this, we applied fMRI-based FC analyses in WT and RAMP1/3 KO mice. The knockout model allowed determining whether observed connectivity changes are mediated by functional amylin receptor signaling. We further aimed at characterizing network dynamics beyond traditional satiation hubs to offer a comprehensive view of amylin's central effects. Unraveling these time-resolved mechanisms is pivotal not only for deepening our understanding of amylin's multifaceted central actions, but also for guiding the development of next-generation anti-obesity therapeutics.

## 2 Results

### 2.1 Peripheral amylin transiently reorganizes brain synchrony as measured by fMRI-based functional connectivity

Resting-state fMRI was continuously acquired from WT (n = 7) and RAMP1/3 KO (n = 7) mice over an 80-minute session. The scanning protocol included a 20-minute baseline, an intraperitoneal (i.p.) saline injection followed by a 30-minute vehicle phase, and a final 30-minute session following i.p. administration of amylin (Fig. 1B). Functional images were parcellated into distinct brain regions (see Supplementary Table 1, Supplementary Fig. S1) to extract regional time series. FC was computed using Pearson's correlation between pairs of regional signals (Fig. 1C). Given the known non-stationarity of FC, we also applied a dynamic functional connectivity (dFC) framework using a sliding-window approach to generate time-resolved connectivity matrices (Fig. 1D).

At baseline, WT mice exhibited robust within-network synchrony (Fig. 1E), whereas KO animals displayed distinct baseline alterations, particularly pronounced in the inferior colliculus, substantia nigra, and nucleus of the lateral lemniscus (Fig. 1F). Building on this, we evaluated strain-specific variability across experimental conditions. Both genotypes showed an increase in FC variance after vehicle injection, but only WT mice displayed a comparable increase following amylin administration (Fig. 1G). While not statistically significant, KO mice showed higher baseline FC variability (0.0843 ± 0.010) than WT mice (0.0728 ± 0.0031, two-sample t-test, t (12) = 1.64, p = 0.12).

To further probe temporal FC dynamics, we segmented the time series into 10-minute intervals and quantified connectivity changes relative to the preceding segment in WT animals. Saline injection did not produce any large-scale reconfiguration (Fig. 1H). In contrast, amylin triggered widespread FC changes within the first 10 min, particularly in the olfactory, amygdalar, thalamic, hypothalamic, and midbrain networks (Fig. 1I). These effects partially subsided in the following 10–20 min, suggesting a transient reorganization trending back toward baseline and coinciding with amylin's biological half-life [38,39].

We next assessed global dFC by calculating, for each ROI, the aggregated sum of its dynamic connectivity with every other ROI over time, providing a measure of coupling strength at the whole-brain level. As expected, regions previously implicated in amylin signaling showed pronounced effects: AP exhibited a decrease, while CeA and paraventricular nucleus of the hypothalamus (PVN) showed increases (Fig. 1J). Unexpectedly, we also observed increased global FC in regions not classically associated with amylin action, such as the anterior dorsal thalamus, visceral (granular insular) cortex, and posterior agranular insula. In contrast, several regions showed minimal or no change (Supplementary Fig. S2), though such stability may reflect opposing or heterogeneous subregional effects obscured in global measures.

### 2.2 Amylin induces broad connectivity shifts beyond canonical feeding pathways

**Figure 2:**
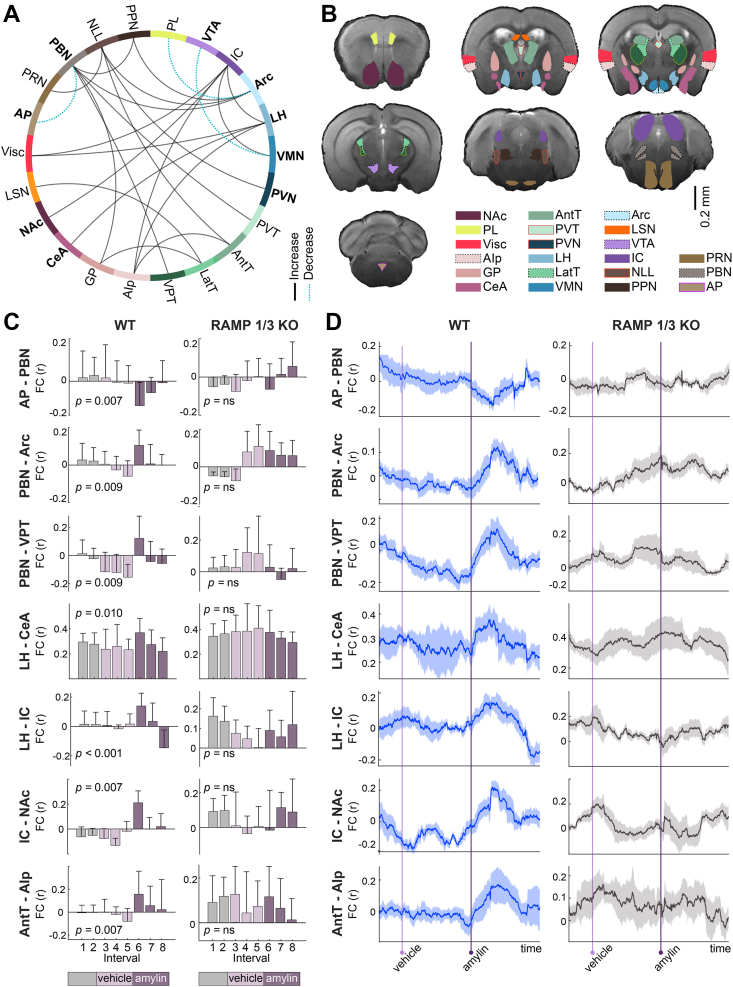
**Temporal evolution of region-specific functional connectivity (FC) alterations induced by amylin. A** Circular connectogram illustrating significant changes in pairwise functional connectivity (FC) in wild-type (WT) mice following peripheral amylin administration (p < 0.01, paired t-test, FDR-corrected). Solid black lines denote increased FC; blue dashed lines indicate decreased FC. **B** Anatomical projection of regions exhibiting significant FC changes, overlaid on a structural reference scan. **C** Time-resolved FC (mean ± SEM) between key region pairs in WT (n = 7) and RAMP1/3 knockout (KO; n = 7) mice, segmented into 10-minute intervals. Distinct temporal profiles reveal rapid and transient modulation in WT animals that is largely absent in KO mice. Statistical significance (paired t-test, FDR-corrected) was assessed by contrasting the first 10-min post-amylin interval with the average of the terminal vehicle and amylin intervals; FDR-corrected p-values are indicated on each plot. **D** Dynamic FC analysis showing temporal evolution of connectivity strength in selected region pairs. PL**,** Prelimbic area; VTA, Ventral tegmental area; IC, Inferior colliculus; ARC, Arcuate hypothalamic nucleus; LH, Lateral hypothalamic area; VMN, Ventromedial hypothalamic nucleus; PVN, Paraventricular hypothalamic nucleus; PVT, Paraventricular nucleus of the thalamus; AntT, Anterior group of the dorsal thalamus; LatT, Lateral group of the dorsal thalamus; VPT, Ventral posterior complex of the thalamus; AIp, Agranular insular area, posterior part; GP, Globus pallidus; CeA, Central amygdalar nucleus, NAc, Nucleus accumbens; LSN, Lateral septal nucleus; AP, Area postrema; PRN, Pontine reticular nucleus; PBN, Parabrachial nucleus; NLL, Nucleus of the lateral lemniscus; PPN, Pedunculopontine nucleus.

Because global FC represents a whole-brain aggregate, opposing subregional effects may cancel each other or be masked by regions not engaged in the response. To reveal these region-specific patterns with higher spatial specificity, we therefore examined pairwise FC between individual regions. Amylin administration altered connectivity not only in centers classically associated with the homeostatic control of eating, but also in regions associated with reward, sensory processing, and visceral integration (Fig. 2A,B, Supplementary Table 2), as determined by paired t-tests contrasting the first 10-min post-amylin window against the mean of the terminal vehicle and amylin intervals with false discovery rate (FDR)-corrected p < 0.01 [2,28,29,40]. In WT mice, notable changes were observed in canonical eating control nodes including the AP, PBN, ARC, PVN, NAc, VTA, and CeA. Additional appetite hubs, such as the paraventricular thalamus (PVT) and lateral hypothalamus (LH), also showed significant FC modulation.

While most amylin-driven effects involved FC increases, select connections were reduced. These included AP–PBN, VTA– ventromedial nucleus of the hypothalamus (VMN), and ARC–prelimbic cortex, highlighting that amylin's effects are region- and direction-specific. Among the earliest responses was a rapid drop in AP–PBN (pFDR = 0.007) connectivity, whereas slower increases were observed in PBN–ARC and PBN–PVT (pFDR = 0.009) (Fig. 2C,D). The LH exhibited a rapid increase in connectivity with the CeA (pFDR = 0.010).

Intriguingly, several noncanonical regions also demonstrated amylin-induced shifts. The inferior colliculus (IC), typically involved in auditory and multisensory processing, increased its connectivity with reward- and energy-relevant nodes including LH, NAc, VMN, and the visceral cortex (Fig. 2C,D; Supplementary Fig. S3). Similarly, the posterior agranular insula (AIp), linked to interoception, showed enhanced connectivity with the anterior dorsal thalamus (AntT), LH, and IC (Supplementary Fig. S4). Other hindbrain structures such as the nucleus of the lateral lemniscus (NLL) and pedunculopontine nucleus (PPN) showed ARC-directed connectivity shifts akin to the PBN, with the NLL also coupling to the AntT and PVN (Supplementary Fig. S5). Notably, these changes were absent in RAMP1/3 KO animals, supporting a receptor-specific action of amylin via the AMY_1/3_R (Fig. 2C,D).

Although amylin-evoked effects observed in WT were not detected in RAMP1/3 KO animals, we observed KO-specific changes in FC (Supplementary Fig. S6). In this genotype, the upper-limb subregion of primary somatosensory cortex exhibited increased FC after amylin administration with the LH, medial thalamus, and midbrain nuclei, including the red nucleus and median raphe nucleus. By contrast, the remaining amylin-associated FC changes in the KO cohort appear injection-related, as they mirrored those seen after vehicle (Supplementary Fig. S6).

### 2.3 Amylin modulates functional brain networks in a strain-specific manner

**Figure 3:**
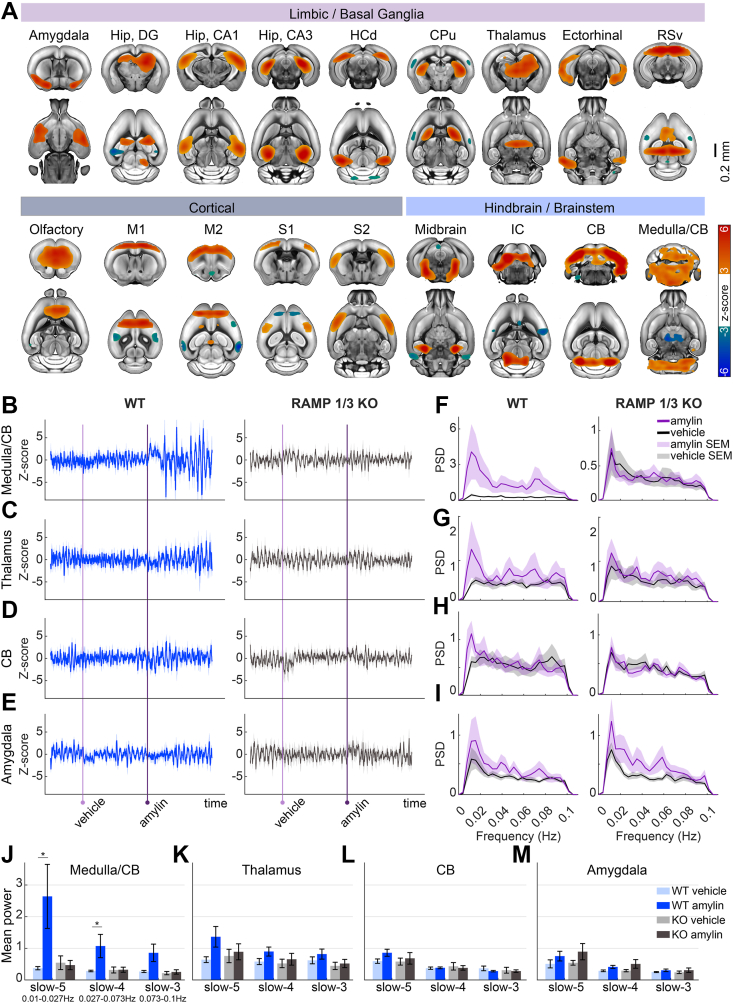
**Amylin modulates intrinsic brain networks and low-frequency oscillatory activity. A** Group-level independent component analysis (ICA) across all animals (n = 14) identified 18 distinct functional networks spanning the cortex, basal ganglia, limbic system, brainstem, and hindbrain. **B-E** Temporal dynamics of representative ICA components following amylin injection in wild-type (WT; n = 7) and RAMP1/3 knockout (KO; n = 7) mice. WT animals exhibited a pronounced amplitude increase in the medulla-dominant component (B), whereas KO mice showed no significant change. **F-I** Power spectral density (PSD) analyses of the ICA component time courses shown in panels b–e under vehicle and amylin conditions. WT mice displayed an amylin-induced increase in low-frequency power (<0.1 Hz), particularly in the medulla/cerebellum network (f; please note the different scale for WT and KO mice), a shift absent in KO animals. **J-M** Band-limited spectral power (mean ± SEM) in slow-5 (0.01–0.027 Hz), slow-4 (0.027–0.073 Hz), and slow-3 (0.073–0.1 Hz) bands for the medulla-dominant (J), thalamic (K), cerebellar (L), and amygdala (M) networks. WT and RAMP1/3 KO mice exhibited comparable power during the vehicle period, whereas amylin selectively increased slow-frequency power in WT, most prominently in the medulla-dominant component. ∗p < 0.05, paired t-tests. Hip, DG, Hippocampus, dentate gyrus; Hip, CA1, Hippocampus, field CA1; Hip, CA3, Hippocampus, field CA3; HCd, Dorsal hippocampal commissure; CPu, Caudoputamen; RSv, Retrosplenial area, ventral part; M1, Primary motor area; M2, Secondary motor area; S1, Primary somatosensory area; S2, Supplemental somatosensory area; IC, Inferior colliculus; CB, Cerebellum.

To evaluate how amylin affects coordinated activity across broader brain systems, we performed independent component analysis (ICA) on the full dataset (80 min functional data from 14 mice, 7 WT, 7 RAMP1/3 KO). This data-driven method identified 20 spatially distinct functional networks, of which 18 were meaningful and spanned cortex, limbic structures, basal ganglia, hindbrain, and brainstem (Fig. 3A).

In WT mice, amylin administration elicited an increase in time-course amplitude in the medulla/cerebellum network, which was not seen in KO mice (Fig. 3B). A milder but notable increase was also observed in the thalamic component (Fig. 3C), while a brief amplitude rise occurred in the cerebellum-specific network (Fig. 3D). In contrast, the amygdala network, despite regional FC changes noted earlier, did not exhibit a clear amplitude shift in either strain (Fig. 3E).

We further explored the frequency structure of these responses using power spectral density (PSD) analysis. In WT mice, amylin robustly increased low-frequency power (<0.1 Hz) in the medulla/cerebellum network, an effect absent in KOs (Fig. 3F). Similar but less pronounced alterations in slow fluctuations (<0.02 Hz) were seen in the thalamic and cerebellar components (Fig. 3G,H). Band-limited analyses across canonical slow-5 (0.01–0.027 Hz), slow-4 (0.027–0.073 Hz), and slow-3 (0.073–0.1 Hz) bands [41] showed highly similar spectra between WT and KO mice during the vehicle period, indicating comparable baseline physiology (Fig. 3J–M). Following amylin injection, however, WT mice exhibited increases in slow-5 power within hindbrain (paired t-test, t (6) = 2.49, p = 0.047) and thalamic (t (6) = 2.27, p = 0.063) networks, whereas KO animals showed no detectable changes (Fig. 3J–M). Several additional networks exhibited elevated low-frequency activity (<0.02 Hz) following the amylin administration, while others remained unaffected (Supplementary Fig. S7).

## 3 Discussion

Understanding how peripheral hormones modulate brain function is essential for uncovering the neural mechanisms that drive complex behaviors such as feeding. Clinically, amylin and its analogs have shown promise for treating metabolic disorders [21,22]. While extensive work has mapped the circuitry regulating hunger and satiation, the precise mechanisms through which amylin alters large-scale brain dynamics remain unclear. Further, most prior studies have relied on static neural readouts that overlook the transient and evolving nature of brain responses. In the present study, we used fMRI-based FC to reveal time-resolved alterations in network synchrony following peripheral amylin administration. This approach avoids assumptions about stimulus timing and hemodynamic response profile, capturing fluctuations in BOLD signals that reflect endogenous circuit reconfiguration. In this context, FC is particularly well suited to resolve the transient circuitry reorganization elicited by amylin, while acknowledging that it cannot fully disentangle direct pharmacological effects from indirect, network-mediated changes. Traditional pharmacological MRI approaches could, in principle, offer complementary voxel-wise amplitude maps, but their reliance on relatively sustained or monotonic BOLD deflections makes them less appropriate for the temporally heterogeneous dynamics that characterize amylin signaling.

In WT mice, peripheral amylin induced a rapid and transient reorganization of functional networks, with most changes emerging within 10 min and beginning to reverse thereafter. These effects were spatially heterogeneous, with distinct onset latencies and durations across regions. Among the earliest modulations, changes occurred between the AP and PBN, consistent with prior evidence indicating the importance of this pathway [27,28,42,43]. As the AP resides outside the blood–brain barrier, this immediate reduction in AP–PBN connectivity likely represents a direct relay of peripheral amylin signals, initiating a cascade of downstream modulation. Notably, AP–PBN FC became more negative relative to baseline, not weaker. After amylin, the absolute FC increased while the sign flipped to negative, indicating stronger but anti-correlated coordination. This polarity shift does not conflict with amylin-evoked c-Fos increases: c-Fos indexes local activation, whereas FC captures temporal coordination that can be inverse-phase. The mechanistic meaning of this anti-correlation remains ambiguous without causal or phase-resolved measures, but it underscores that amylin strengthens, rather than silences, AP–PBN communication. From the PBN, changes propagated to thalamic and hypothalamic structures, including the ARC and LH, nodes with well-established roles in metabolic sensing and satiation signaling. The anatomical specificity of these responses validates prior work identifying amylin-responsive sites with complementary methods [44], and our dynamic FC analysis offers new evidence that the AP is a primary gateway for amylin's central effects.

A recent study using static FC reported sex-specific effects of amylin on brain networks [45]. While our cohort was male-only, we identified convergent changes in the ARC, PBN, NAc, and LH; in contrast, we did not observe the retrosplenial cortex alterations reported previously. However, it is important to note that static connectivity frameworks are limited by their inability to resolve temporally evolving responses. By collapsing fluctuations into a single summary metric, such approaches risk conflating transient or sequential changes and may mischaracterize dynamic neuromodulatory processes. These limitations make static FC analyses poorly suited to capturing the true temporal extent and specificity of amylin's effects on brain networks.

Interestingly, we did not observe significant connectivity responses in the NTS or BNST, despite previous evidence of their involvement in amylin signaling [31,46]. This may reflect signal components too subtle or transient to be captured at our current temporal resolution, or neural dynamics that are not well-indexed by BOLD fluctuations. It is also possible that these regions require longer integration windows to reveal their engagement by amylin.

The near-complete absence of amylin-induced connectivity changes in RAMP1/3 KO mice confirms the necessity of intact amylin receptor signaling for large-scale network modulation. This observation is consistent with prior c-Fos mapping [25] and highlights the functional impact of receptor availability on brain-wide modulation. Notably, baseline FC patterns in KO animals also diverged from WT mice, which may suggest long-term circuit reorganization arising from disrupted amylin signaling. These changes may also reflect compensatory adaptations or altered synaptic wiring in the absence of receptor-mediated feedback. Previous studies showing reduced AgRP neuronal projections in PVN of KO animals support this view [33], and similar alterations may extend to other neuronal subtypes, potentially influencing the brain architecture.

Among the FC differences between WT and RAMP1/3 KO mice, the IC and NLL emerged as functional hubs with distinct response profiles. Although traditionally classified as auditory centers, both regions participate in multisensory integration and increasingly show relevance for adaptive behavioral regulation [47,48]. Recent findings implicating the NLL in neuropeptide FF signaling and energy balance [49,50] suggest a broader role in metabolic control. In WT animals, increased coupling between these sensory hubs and core satiation regions may support the integration of external cues with internal metabolic states. Conversely, their disrupted connectivity in KO mice implies a breakdown in sensory-metabolic alignment, possibly contributing to impaired energy homeostasis.

Another noncanonical node, the AIp, also exhibited robust amylin-driven changes. Although not historically linked to feeding control, the AIp is known for integrating interoceptive, visceral, and emotional signals [[51], [52], [53]]. Its increased connectivity with the LH, AntT, and IC suggests that amylin may influence the translation of visceral sensations into motivational or behavioral outputs. The AIp may thus act as a bridge between internal physiological states (e.g., satiation) and external sensory cues (e.g., food availability).

At the network level, ICA revealed distributed spatial patterns that were modulated by amylin. The medulla-dominant component showed the strongest amplitude response in WT mice, consistent with this region's high receptor density and central role in autonomic and visceral integration [25,27,31]. Furthermore, power spectral analysis revealed that amylin altered low-frequency oscillations in several components. These slow rhythms are linked to both vascular regulation and baseline neural synchrony, raising the possibility that amylin influences not only the spatial topology but also the temporal architecture of functional networks.

Despite these insights, several limitations must be acknowledged. First, fMRI-based FC reflects an indirect measure of neuronal activity. Future work integrating calcium imaging, electrophysiology, or cellular-resolution optical techniques will be essential in studying the newly emerging amylin hubs to confirm and refine our findings. Second, the use of anesthetized animals may suppress certain neural dynamics or behavioral correlates. Third, whole-body RAMP1/3 deletion results in baseline network differences that could interact with or mask amylin-evoked effects. This includes the possibility of genotype-dependent physiological differences, such as altered vascular tone or impaired vasodilatory signaling [54], that may further shape the observed responses. Fourth, although aliasing of high-frequency physiological rhythms is a recognized limitation in rodent rs-fMRI [55], the matched spectral structure across genotypes during the vehicle condition makes it unlikely that these factors underlie the observed amylin-specific differences. Fifth, the spatial resolution of fMRI limits the detection of small or anatomically complex nuclei that may participate in key regulatory processes. Finally, our study was conducted in male mice only, which reduces sex-dependent variability but limits generalizability. Future work including both sexes will be essential to determine whether the network dynamics identified here extend to female animals, particularly given prior reports of sex-specific amylin effects [45].

Future studies should incorporate complementary neuroimaging and behavioral paradigms to further dissect how amylin shapes feeding-related circuitry. Since endogenous amylin is secreted in pulses after meals, repeated activation may induce long-term network reorganization not captured by a single-dose paradigm. Longitudinal studies will be needed to distinguish acute effects from chronic adaptations, e.g. reflecting differences in baseline amylin concentrations that depend on the state of obesity [56,57] which may add additional effects that could not be captured here. Additionally, exploring interactions between amylin and other hormones such as leptin, insulin, or ghrelin may uncover synergistic mechanisms that drive flexible, context-dependent regulation of appetite. It will also be important to determine how long-acting amylin analogues, such as cagrilintide or eloralintide, reshape network dynamics relative to native amylin, as their prolonged pharmacokinetics may engage distinct or more sustained circuit mechanisms. Direct comparison within a similar dynamic fMRI framework could reveal neural features that underlie their enhanced therapeutic efficacy. These insights are especially relevant as combinatorial pharmacological strategies such as dual amylin/GLP-1 receptor agonists are now showing superior outcomes for weight loss and glycemic control relative to monotherapy [21,22]. Understanding how these agents remodel the brain's feeding networks could thus inform a new generation of targeted treatments for obesity and related metabolic diseases.

## 4 Methods

### 4.1 Animal models

129S2/SvPasorIRj mice wild-type (WT, n = 8, 10–12 weeks old, male) and RAMP 1/3 knock out (KO, n = 8, 10–12 weeks old, male) issued from our in-house colony were used in this study (founder animals were kindly provided by Prof. Kathleen Caron, University of North Carolina, USA). The mice were fed with a standard chow diet (Diet n°3436, Provimi Kliba AG, Kaiseraugst, Switzerland) with free access to food (except during periods of food deprivation as explained below) and water throughout the experiments. The diet had an energy content of 3.15 kcal/g, with 65.4% of energy coming from carbohydrates, 12.3% of fat, and 22.4% of protein. The animals were handled and acclimated to their housing conditions for at least one week before the start of the experiments. Mouse housing, handling, and experimentation were performed in accordance with the Swiss Federal Act on Animal Protection and were approved by the Cantonal Veterinary Office Zurich.

### 4.2 In vivo imaging

Animals were food-deprived for 2 h prior to imaging during the light phase. Anesthesia was induced with 4% isoflurane in air and maintained at <1.5% during preparatory procedures including head fixation, magnetic homogeneity adjustment, shimming, and reference pulse acquisition. Subsequently, a 0.05 mg/kg intravenous bolus of medetomidine (Domitor, medetomidine hydrochloride; Pfizer Pharmaceuticals, Sandwich, UK) was administered. Five minutes post-injection, isoflurane was reduced to 0.7% to facilitate sedation, and a continuous medetomidine infusion (0.1 mg/kg/h) was initiated 10 min after the bolus. Thirty minutes post-medetomidine injection and following a structural MRI scan, fMRI data acquisition commenced and continued for 80 min per animal. At 20 min into the acquisition, animals received an intraperitoneal vehicle injection, and at 50 min, they were administered an intraperitoneal injection of amylin (500 μg/kg, 4,030,201, Bachem AG, Bubendorf, Switzerland; reconstituted in sterile 0.9% saline). All animals were naïve to experimental drugs and tests prior to the study. To minimize potential confounders, scan order across days was alternated between genotypes, and animals from different cages were imaged in mixed sequence. All mice underwent the same experimental paradigm, ensuring consistent timing across groups. Respiration and rectal temperature were continuously monitored, and body temperature was maintained at 37 °C using an MR-compatible, feedback-controlled water heating system.

### 4.3 MRI data acquisition

The MRI acquisitions were performed using a 7.0 T MRI system (Biospec; 70/16 Bruker BioSpin, Ettlingen, Germany) equipped with a cryogenic quadrature radio frequency (RF) surface probe (CryoProbe; Bruker BioSpin AG, Fällanden, Switzerland). Anatomical scans were acquired using a T2-weighted rapid acquisition with relaxation enhancement (RARE) sequence with the following parameters: field of view (FOV) = 20 × 10 mm^2^, matrix dimension (MD) = 256 × 128, slice thickness = 0.4 mm, repetition time (TR) = 3000 ms, echo time (TE) = 15 ms, RARE factor = 8, and number of averages (NA) = 6. Functional MRI data were captured using a gradient echo–echo planar imaging (GE-EPI) sequence: FOV = 20 × 10 mm^2^, MD = 90 × 50, in-plane voxel size = 222 × 200 μm^2^, slice thickness = 0.4 mm, flip angle (FA) = 60°, TR = 1000 ms, TE = 15 ms, and NA = 1. The scan was repeated 4800 times over an 80-minute duration for each session. Prior to functional imaging, B0 field maps were acquired and used to optimize local field homogeneity.

### 4.4 Functional MRI data preprocessing

Functional MRI datasets were preprocessed using SPM12 (Wellcome Trust Centre for Neuroimaging, London, UK), the CONN toolbox [58], and custom MATLAB scripts. Data from one wild-type and one knockout animal were excluded due to ghosting artifacts. Each subject's anatomical and functional images were co-registered and subsequently normalized to the Allen Mouse Brain Common Coordinate Framework (CCF) [59] using both linear and nonlinear transformations. The functional images were resampled to an isotropic voxel size of 0.2 × 0.2 × 0.2 mm^3^, realigned for motion correction, and spatially smoothed with a 0.6 mm full-width-at-half-maximum (FWHM) Gaussian kernel. Temporal preprocessing included detrending and bandpass filtering in the 0.008–0.1 Hz frequency range. To minimize non-neuronal signal contributions, signals from white matter (WM), cerebrospinal fluid (CSF), and six motion parameters (three translation, three rotation) were regressed out. Finally, the preprocessed data were segmented into 10-minute intervals for each acquisition for subsequent analyses. During data preprocessing and statistical analysis, datasets were anonymized and coded by genotype to ensure blinding of the analyst. Group identities were only revealed after all analyses were completed.

### 4.5 Functional connectivity analysis

Functional connectivity was quantified from denoised fMRI time-series using regions of interest (ROIs) defined by the Allen Common Coordinate Framework (Supplementary Table 1). Each functional scan was segmented into consecutive 10-minute intervals, yielding 2 intervals for baseline and 3 intervals each for the vehicle and amylin conditions (Fig. 1B). Mean time-series were extracted from each ROI, and ROI-to-ROI connectivity matrices were generated by computing pairwise Pearson's correlation coefficients (r) for each interval (Fig. 1C–F). To assess time-resolved changes in WT mice, difference matrices were calculated by subtracting the connectivity matrix of the preceding interval from that of the subsequent interval (Fig. 1H,I).

Dynamic functional connectivity (dFC) was evaluated using a sliding-window approach [60]. Pearson's correlation coefficients were computed within a 300-second window that was shifted by one TR (1 s), resulting in 4500 dFC matrices per animal. Global FC for each ROI was quantified by summing its correlations with all other regions (Fig. 1J). To isolate the effects of amylin in WT mice, the first 10-minute interval following amylin injection was compared to the average connectivity derived from the last 10-minute intervals of both the vehicle and amylin conditions. This contrast was designed to disentangle amylin-specific effects from time-dependent changes in FC, as indicated by the global FC results. Correlation values were Fisher's z-transformed prior to statistical analysis. Statistical significance was assessed using Student's t-tests with False Discovery Rate (FDR) correction (p < 0.05). FC alterations were plotted for each 10-minute interval, and injection-mediated effects that appeared following both vehicle and amylin administration were excluded from further analysis. ROI pairs exhibiting significant amylin-induced changes in connectivity were subsequently visualized using a circular connectogram (Fig. 2A,B). Additionally, FC and dFC curves for both WT and RAMP 1/3 KO strains were presented for region pairs (Fig. 2C,D).

### 4.6 Independent component analysis (ICA)

Group-level (n = 14, 7 WT, 7 RAMP 1/3 KO) independent component analysis (ICA) [61] was carried out using the CONN toolbox with 20 components. Spatially independent components along with their corresponding time courses were estimated and meaningful networks were identified (18/20 components) by visual inspection ensuring bilateral patterns matching established resting-state network templates (Fig. 3a). Components corresponding to physiological noise or artifacts were excluded from further analysis.

ICA time courses were then segmented according to experimental conditions (vehicle versus amylin) to assess potential neuromodulatory effects. Specifically, we examined amylin-induced changes by comparing the time courses between the vehicle and amylin conditions within each genotype (Fig. 3B–E). For a quantitative assessment, power spectral density (PSD) estimates of the ICA time courses were calculated using Welch's method.

## CRediT authorship contribution statement

**Irmak Gezginer:** Writing – original draft, Visualization, Methodology, Investigation, Formal analysis, Data curation. **Giulia Mazzini:** Writing – review & editing, Investigation, Data curation. **Christelle Le Foll:** Writing – review & editing, Project administration, Methodology, Conceptualization. **Diana Kindler:** Data curation. **Thomas A. Lutz:** Writing – review & editing, Supervision, Resources, Funding acquisition, Conceptualization. **Daniel Razansky:** Writing – review & editing, Supervision, Project administration, Funding acquisition, Conceptualization.

## Code availability

The code that support the findings of this study are available from the corresponding author upon request.

## Funding

The authors acknowledge support from the 10.13039/100000001Swiss National Science Foundation grant 207763 and 10.13039/100000001Swiss National Science Foundation grant 192757.

## Declaration of competing interest

The authors declare the following financial interests/personal relationships which may be considered as potential competing interests: Thomas Lutz reports financial support was provided by Swiss National Science Foundation. Daniel Razansky reports was provided by Swiss National Science Foundation. If there are other authors, they declare that they have no known competing financial interests or personal relationships that could have appeared to influence the work reported in this paper.

## Data Availability

The data that support the findings of this study are available from the corresponding authors upon request. All data supporting the findings of this study are found within the paper and its Supplementary Information.

## References

[1] Xin J., Tingting Q., Li L., Rilei Y., Xiguang C., Changgui L. (2023). Pathophysiology of obesity and its associated diseases. Acta Pharm Sin B.

[2] Ahima R.S., Antwi D.A. (2008). Brain regulation of appetite and satiety. Endocrinol Metab Clin N Am.

[3] Scrocchi L.A., Brown T.J., Maclusky N., Brubaker P.L., Auerbach A.B., Joyner A.L. (1996). Glucose intolerance but normal satiety in mice with a null mutation in the glucagon–like peptide 1 receptor gene. Nat Med.

[4] Sarika A. (2006). Anubhuti. Role of neuropeptides in appetite regulation and obesity – a review. Neuropeptides.

[5] Müller T.D., Nogueiras R., Andermann M.L., Andrews Z.B., Anker S.D., Argente J. (2015). Ghrelin. Mol Metabol.

[6] Ahima R.S., Prabakaran D., Mantzoros C., Qu D., Lowell B., Maratos-Flier E. (1996). Role of leptin in the neuroendocrine response to fasting. Nature.

[7] Alcantara I.C., Tapia A.P.M., Aponte Y., Krashes M.J. (2022). Acts of appetite: neural circuits governing the appetitive, consummatory, and terminating phases of feeding. Nat Metab.

[8] Fu O., Iwai Y., Narukawa M., Ishikawa A.W., Ishii K.K., Murata K. (2019). Hypothalamic neuronal circuits regulating hunger-induced taste modification. Nat Commun.

[9] Schaeffer M., Langlet F., Lafont C., Molino F., Hodson D.J., Roux T. (2013). Rapid sensing of circulating ghrelin by hypothalamic appetite-modifying neurons. Proc Natl Acad Sci USA.

[10] Gina M.L., Young-Hwan J., Rebecca L.L., Gwendolyn W.L., Hongyan Y., Jason G.B. (2009). Leptin acts via leptin receptor-expressing lateral hypothalamic neurons to modulate the mesolimbic dopamine system and suppress feeding. Cell Metab.

[11] Edward H.N., Caitlin MVander W., Gillian A.M., Kara N.P., Romy W., Christopher A.L. (2016). Inhibitory input from the lateral hypothalamus to the ventral tegmental area disinhibits dopamine neurons and promotes behavioral activation. Neuron.

[12] Eoin C.O.C., Yves K., Sandrine L., Masaya H., Vincent P., Clément R. (2015). Accumbal D1R neurons projecting to lateral hypothalamus authorize feeding. Neuron.

[13] Gaziano I., Corneliussen S., Biglari N., Neuhaus R., Shen L., Sotelo-Hitschfeld T. (2022). Dopamine-inhibited POMCDrd2+ neurons in the ARC acutely regulate feeding and body temperature. JCI Insight.

[14] Labouèbe G., Liu S., Dias C., Zou H., Wong J.C., Karunakaran S. (2013). Insulin induces long-term depression of ventral tegmental area dopamine neurons via endocannabinoids. Nat Neurosci.

[15] Hommel J.D., Trinko R., Sears R.M., Georgescu D., Liu Z.W., Gao X.B. (2006). Leptin receptor signaling in midbrain dopamine neurons regulates feeding. Neuron.

[16] Lutz T.A. (2010). The role of amylin in the control of energy homeostasis. Am J Physiol Regul Integr Comp Physiol.

[17] Lutz T.A., Geary N., Szabady M.M., Del Prete E., Scharrer E. (1995). Amylin decreases meal size in rats. Physiol Behav.

[18] Lutz T.A. (2009). Frontiers in eating and weight regulation [Internet].

[19] Thomas A.L. (2005). Pancreatic amylin as a centrally acting satiating hormone. Curr Drug Targets.

[20] Lau D.C.W., Erichsen L., Francisco A.M., Satylganova A., le Roux C.W., McGowan B. (2021). Once-weekly cagrilintide for weight management in people with overweight and obesity: a multicentre, randomised, double-blind, placebo-controlled and active-controlled, dose-finding phase 2 trial. Lancet.

[21] Garvey W.T., Blüher M., Osorto Contreras C.K., Davies M.J., Winning Lehmann E., Pietiläinen K.H. (2025). Coadministered cagrilintide and semaglutide in adults with overweight or obesity. N Engl J Med.

[22] Davies M.J., Bajaj H.S., Broholm C., Eliasen A., Garvey W.T., le Roux C.W. (2025). Cagrilintide-semaglutide in adults with overweight or obesity and type 2 diabetes. N Engl J Med.

[23] McLatchie L.M., Fraser N.J., Main M.J., Wise A., Brown J., Thompson N. (1998). RAMPs regulate the transport and ligand specificity of the calcitonin-receptor-like receptor. Nature.

[24] Coester B., Pence S.W., Arrigoni S., Boyle C.N., Le Foll C., Lutz T.A. (2020). RAMP1 and RAMP3 differentially control amylin's effects on food intake, glucose and energy balance in male and female mice. Neuroscience.

[25] Skovbjerg G., Roostalu U., Hansen H.H., Lutz T.A., Le Foll C., Salinas C.G. (2021). Whole-brain mapping of amylin-induced neuronal activity in receptor activity-modifying protein 1/3 knockout mice. Eur J Neurosci.

[26] Carvas A.O., Leuthardt A., Kulka P., Lommi G., Hassan S., Coester B. (2025). Cagrilintide lowers bodyweight through brain amylin receptors 1 and 3. EBioMedicine.

[27] Braegger F.E., Asarian L., Dahl K., Lutz T.A., Boyle C.N. (2014). The role of the area postrema in the anorectic effects of amylin and salmon calcitonin: behavioral and neuronal phenotyping. Eur J Neurosci.

[28] Boccia L., Gamakharia S., Coester B., Whiting L., Lutz T.A., Le Foll C. (2020). Amylin brain circuitry. Peptides.

[29] Hankir M.K., Le Foll C. (2025). Central nervous system pathways targeted by amylin in the regulation of food intake. Biochimie.

[30] Sexton P.M., Paxinos G., Kenney M.A., Wookey P.J., Beaumont K. (1994). In vitro autoradiographic localization of amylin binding sites in rat brain. Neuroscience.

[31] Lutz T.A., Mollet A., Rushing P.A., Riediger T., Scharrer E. (2001). The anorectic effect of a chronic peripheral infusion of amylin is abolished in area postrema/nucleus of the solitary tract (AP/NTS) lesioned rats. Int J Obes Relat Metab Disord.

[32] Mietlicki-Baase E.G., Reiner D.J., Cone J.J., Olivos D.R., McGrath L.E., Zimmer D.J. (2015). Amylin modulates the mesolimbic dopamine system to control energy balance. Neuropsychopharmacology.

[33] Lutz T.A., Coester B., Whiting L., Dunn-Meynell A.A., Boyle C.N., Bouret S.G. (2018). Amylin selectively signals onto POMC neurons in the arcuate nucleus of the hypothalamus. Diabetes.

[34] Zakariassen H.L., John L.M., Lykkesfeldt J., Raun K., Glendorf T., Schaffer L. (2020). Salmon calcitonin distributes into the arcuate nucleus to a subset of NPY neurons in mice. Neuropharmacology.

[35] Su Z., Alhadeff A.L., Betley J.N. (2017). Nutritive, post-ingestive signals are the primary regulators of AgRP neuron activity. Cell Rep.

[36] Van Den Heuvel M.P., Pol H.E.H. (2010). Exploring the brain network: a review on resting-state fMRI functional connectivity. Eur Neuropsychopharmacol.

[37] Gezginer I., Chen Z., Yoshihara H.A.I., Dean-Ben X.L., Zerbi V., Razansky D. (2024). Concurrent optoacoustic tomography and magnetic resonance imaging of resting-state functional connectivity in the mouse brain. Nat Commun.

[38] Vine W., Blase E., Koda J., Young A. (1998). Plasma amylin concentrations in fasted and fed rats quantified by a monoclonal immunoenzymometric assay. Horm Metab Res.

[39] Young A. (2005). Tissue expression and secretion of amylin. Adv Pharmacol.

[40] Lora K.H., Daniel D.L. (2017). An appetite for life: brain regulation of hunger and satiety. Curr Opin Pharmacol.

[41] Zuo X.N., Di Martino A., Kelly C., Shehzad Z.E., Gee D.G., Klein D.F. (2010). The oscillating brain: complex and reliable. Neuroimage.

[42] Becskei C., Grabler V., Edwards G.L., Riediger T., Lutz T.A. (2007). Lesion of the lateral parabrachial nucleus attenuates the anorectic effect of peripheral amylin and CCK. Brain Res.

[43] Boccia L., Le Foll C., Lutz T.A. (2020). Noradrenaline signaling in the LPBN mediates amylin's and salmon calcitonin's hypophagic effect in male rats. FASEB J.

[44] Dunn-Meynell A.A., Le Foll C., Johnson M.D., Lutz T.A., Hayes M.R., Levin B.E. (2016). Endogenous VMH amylin signaling is required for full leptin signaling and protection from diet-induced obesity. Am J Physiol Regul Integr Comp Physiol.

[45] Arefin T.M., Börchers S., Olekanma D., Cramer S.R., Sotzen M.R., Zhang N. (2025). Sex-specific signatures of GLP-1 and amylin on resting state brain activity and functional connectivity in awake rats. Neuropharmacology.

[46] Abegg K., Hermann A., Boyle C.N., Bouret S.G., Lutz T.A., Riediger T. (2017). Involvement of amylin and leptin in the development of projections from the area postrema to the nucleus of the solitary tract. Front Endocrinol.

[47] Gruters K.G., Groh J.M. (2012). Sounds and beyond: multisensory and other non-auditory signals in the inferior colliculus. Front Neural Circ.

[48] Pagella S., Deussing J.M., Kopp-Scheinpflug C. (2021). Expression patterns of the neuropeptide urocortin 3 and its receptor CRFR2 in the mouse central auditory system. Front Neural Circ.

[49] Higo S., Kanaya M., Ozawa H. (2021). Expression analysis of neuropeptide FF receptors on neuroendocrine-related neurons in the rat brain using highly sensitive in situ hybridization. Histochem Cell Biol.

[50] Lin Y.T., Wu K.H., Jhang J.J., Jhang J.L., Yu Z., Tsai S.C. (2024). Hypothalamic NPFFR2 attenuates central insulin signaling and its knockout diminishes metabolic dysfunction in mouse models of diabetes mellitus. Clin Nutr.

[51] Shiratori R., Yokoi T., Kinoshita K., Xue W., Sasaki T., Kuga N. (2024). The posterior Insular cortex is necessary for feeding-induced jejunal myoelectrical activity in Male rats. Neuroscience.

[52] Wu Y., Chen C., Chen M., Qian K., Lv X., Wang H. (2020). The anterior insular cortex unilaterally controls feeding in response to aversive visceral stimuli in mice. Nat Commun.

[53] Gehrlach D.A., Dolensek N., Klein A.S., Roy Chowdhury R., Matthys A., Junghänel M. (2019). Aversive state processing in the posterior insular cortex. Nat Neurosci.

[54] Tsujikawa K., Yayama K., Hayashi T., Matsushita H., Yamaguchi T., Shigeno T. (2007). Hypertension and dysregulated proinflammatory cytokine production in receptor activity-modifying protein 1-deficient mice. Proc Natl Acad Sci USA.

[55] Pais-Roldán P., Biswal B., Scheffler K., Yu X. (2018). Identifying respiration-related aliasing artifacts in the rodent resting-state fMRI. Front Neurosci.

[56] Boyle C.N., Rossier M.M., Lutz T.A. (2011). Influence of high-fat feeding, diet-induced obesity, and hyperamylinemia on the sensitivity to acute amylin. Physiol Behav.

[57] Pieber T.R., Roitelman J., Lee Y., Luskey K.L., Stein D.T. (1994). Direct plasma radioimmunoassay for rat amylin-(1-37): concentrations with acquired and genetic obesity. Am J Physiol.

[58] Whitfield-Gabrieli S., Nieto-Castanon A. (2012). Conn: a functional connectivity toolbox for correlated and anticorrelated brain networks. Brain Connect.

[59] Wang Q.X., Ding S.L., Li Y., Royall J., Feng D., Lesnar P. (2020). The Allen mouse brain common coordinate framework: a 3D reference atlas. Cell.

[60] Hutchison R.M., Womelsdorf T., Allen E.A., Bandettini P.A., Calhoun V.D., Corbetta M. (2013). Dynamic functional connectivity: promise, issues, and interpretations. Neuroimage.

[61] McKeown M.J., Hansen L.K., Sejnowsk T.J. (2003). Independent component analysis of functional MRI: what is signal and what is noise?. Curr Opin Neurobiol.

