# Supplementary material for: Pancreatic amylin dynamically reconfigures distributed brain networks governing appetite regulation in mice
Source: Mol Metab. 2025 Dec 22;103:102313. doi: 10.1016/j.molmet.2025.102313 (PMC12808607; doi:10.1016/j.molmet.2025.102313)
Supplement: Multimedia component 1 [file mmc1.docx]

**Supplementary Information**

**Pancreatic amylin dynamically reconfigures distributed brain networks governing appetite regulation in mice**

Irmak Gezginer^1,2^, Giulia Mazzini^3^, Christelle Le Foll^3^, Diana Kindler^2^, Thomas A. Lutz^3,*^, Daniel Razansky^1,2,*^

^1^Institute for Biomedical Engineering and Institute of Pharmacology and Toxicology, Faculty of Medicine, University of Zurich, Switzerland

^2^Institute for Biomedical Engineering, Department of Information Technology and Electrical Engineering, ETH Zurich, Switzerland

^3^Institute of Veterinary Physiology, Vetsuisse Faculty, University of Zurich, Zurich, Switzerland

^*^Correspondence to: tomlutz@vetphys.uzh.ch or daniel.razansky@uzh.ch


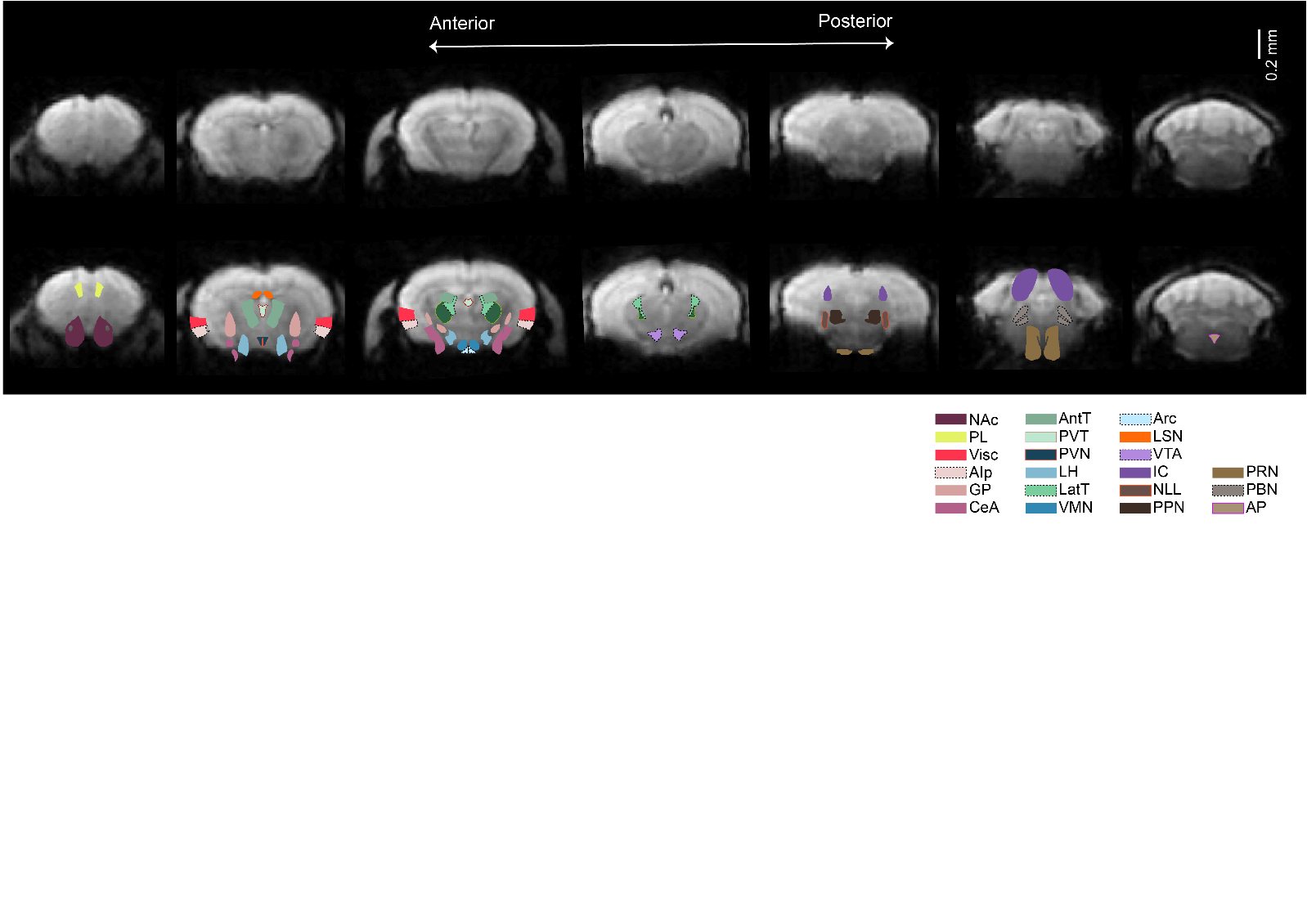


**Supplementary Figure S1.** Raw functional MRI echo-planar imaging (EPI) data from a representative mouse, shown as distributed coronal slices (top), and the same slices overlaid with atlas-derived region boundaries following registration to the Allen Mouse Brain Atlas (bottom). Region definitions correspond to those used in Fig. 2b.

**
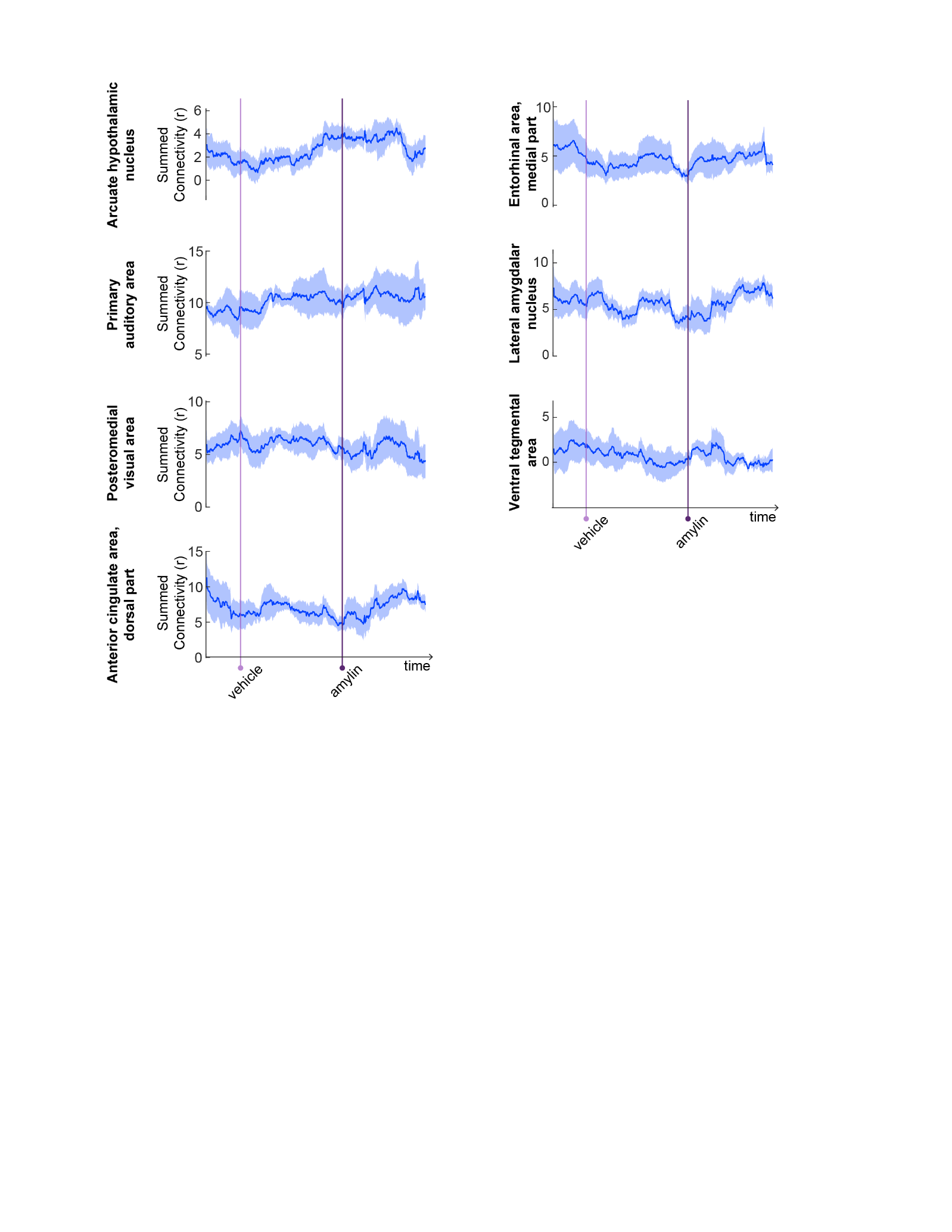
**

**Supplementary Figure S2.** Regional changes in overall functional connectivity (FC). The sum of FC (mean ± SEM) to all other regions following amylin injection, visualized for diverse regions. The FC in the shown regions did not exhibit visible amylin-induced alterations in their global FC.


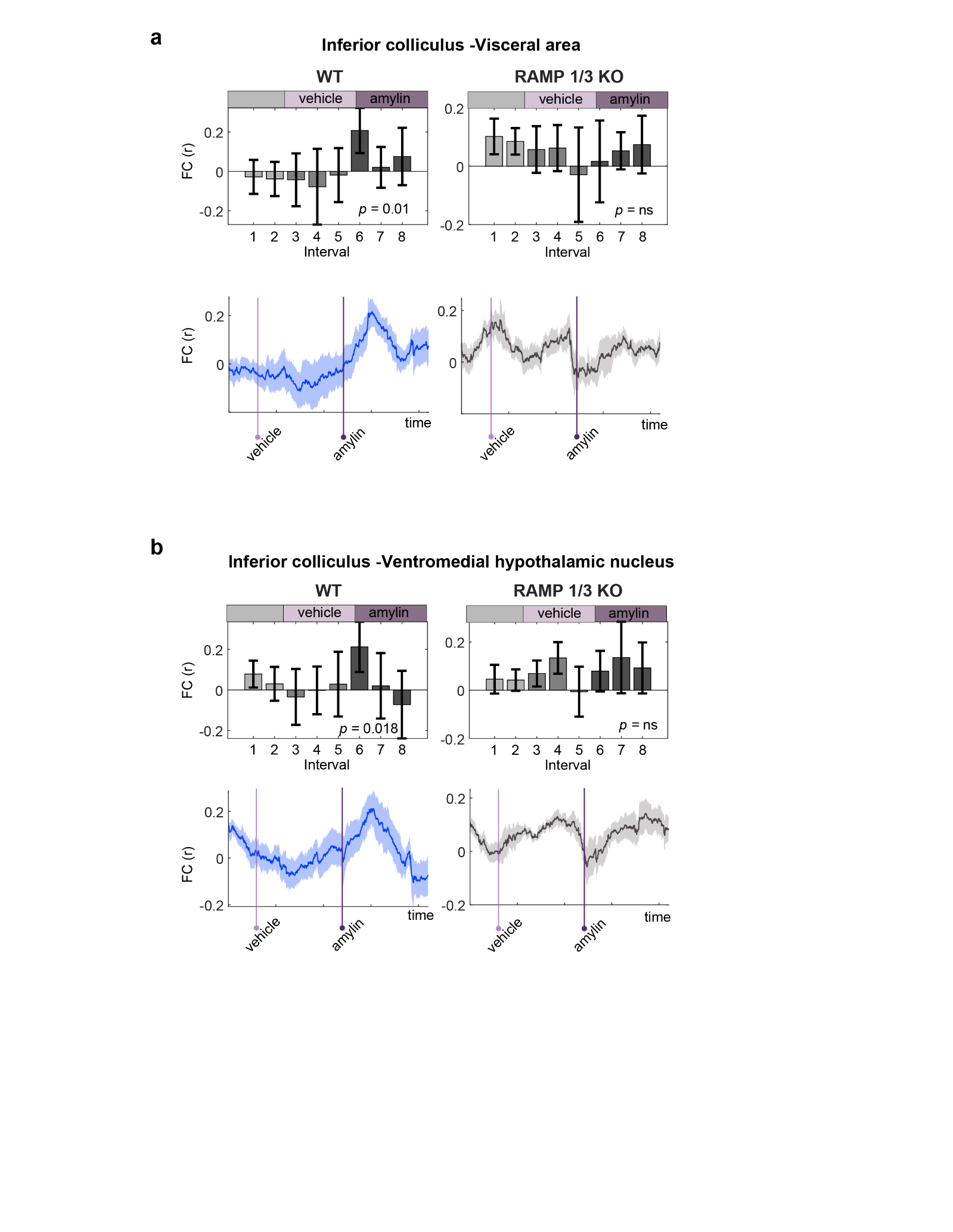


**Supplementary Figure S3.** Time-resolved evolution of mean FC (± SEM) between inferior colliculus to visceral area (a) and ventromedial hypothalamic nucleus (b) for wild-type (WT) (left) and RAMP 1/3 knockout (KO) (right) mice. Data are segmented into 10-minute intervals for the bar plots. Corresponding p values are given on each plot. Lower plots detail dynamic FC of the same region-pairs.


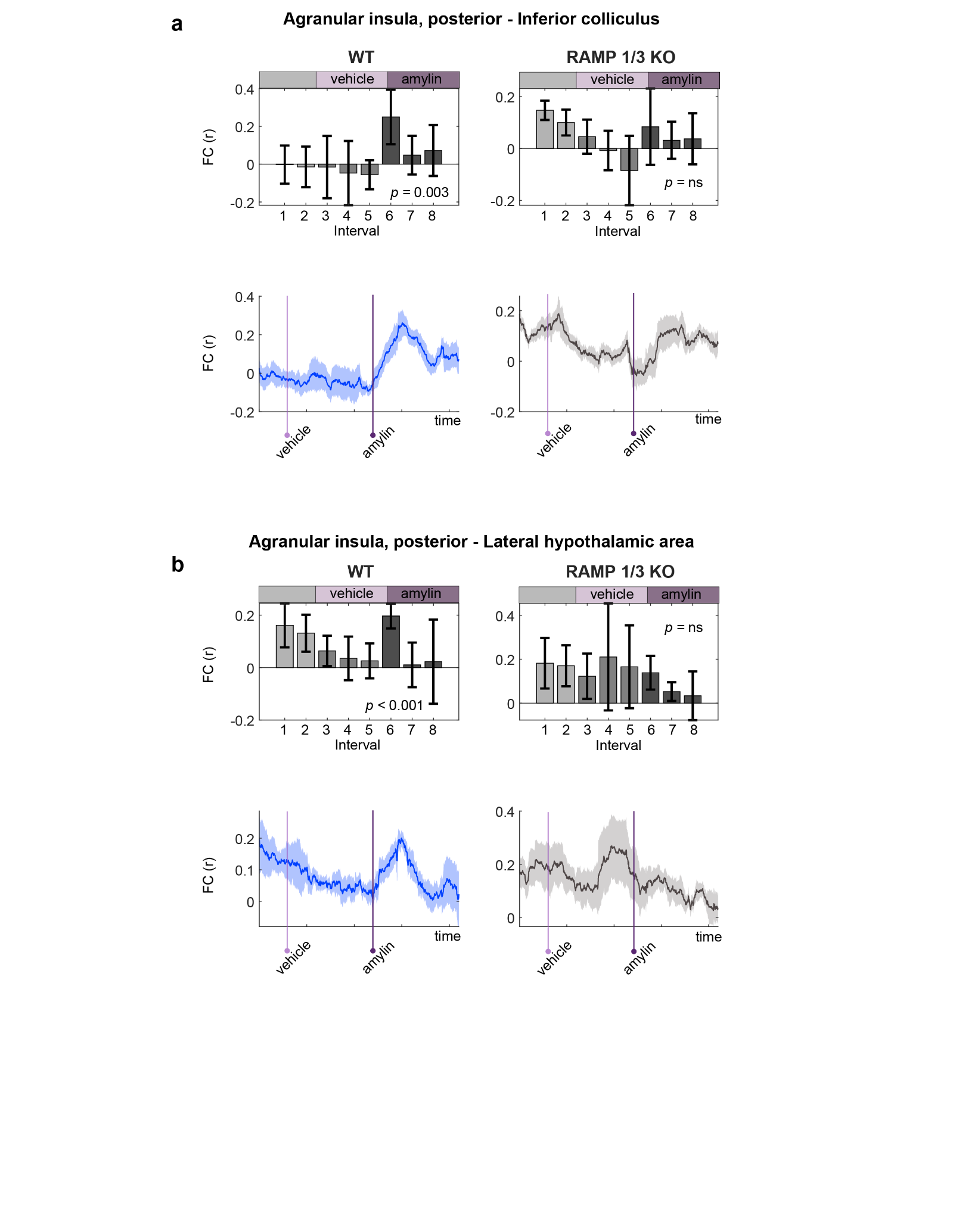


**Supplementary Figure S4.** Time-resolved evolution of mean FC (± SEM) between posterior agranular insula to inferior colliculus (a) and lateral hypothalamic area (b) for wild-type (WT) (left) and RAMP 1/3 knockout (KO) (right) mice. Data are segmented into 10-minute intervals for the bar plots. Corresponding p values are given on each plot. Lower plots detail dynamic FC of the same region-pairs.


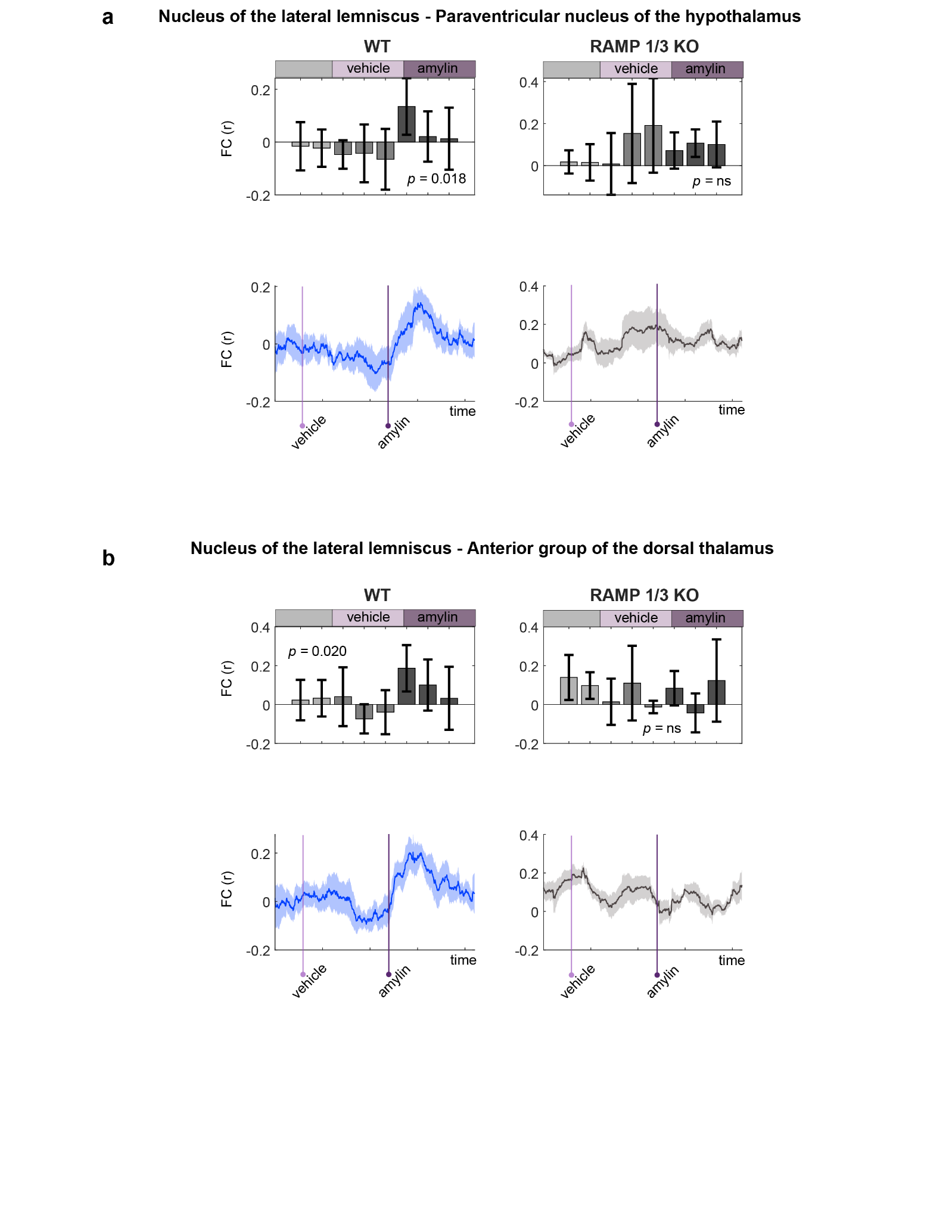


**Supplementary Figure S5.** Time-resolved evolution of mean FC (± SEM) between nucleus of lateral lemniscus to paraventricular nucleus of hypothalamus (a) and anterior group of dorsal thalamus (b) for wild-type (WT) (left) and RAMP 1/3 knockout (KO) (right) mice. Data are segmented into 10-minute intervals for the bar plots. Corresponding p values are given on each plot. Lower plots detail dynamic FC of the same region-pairs.


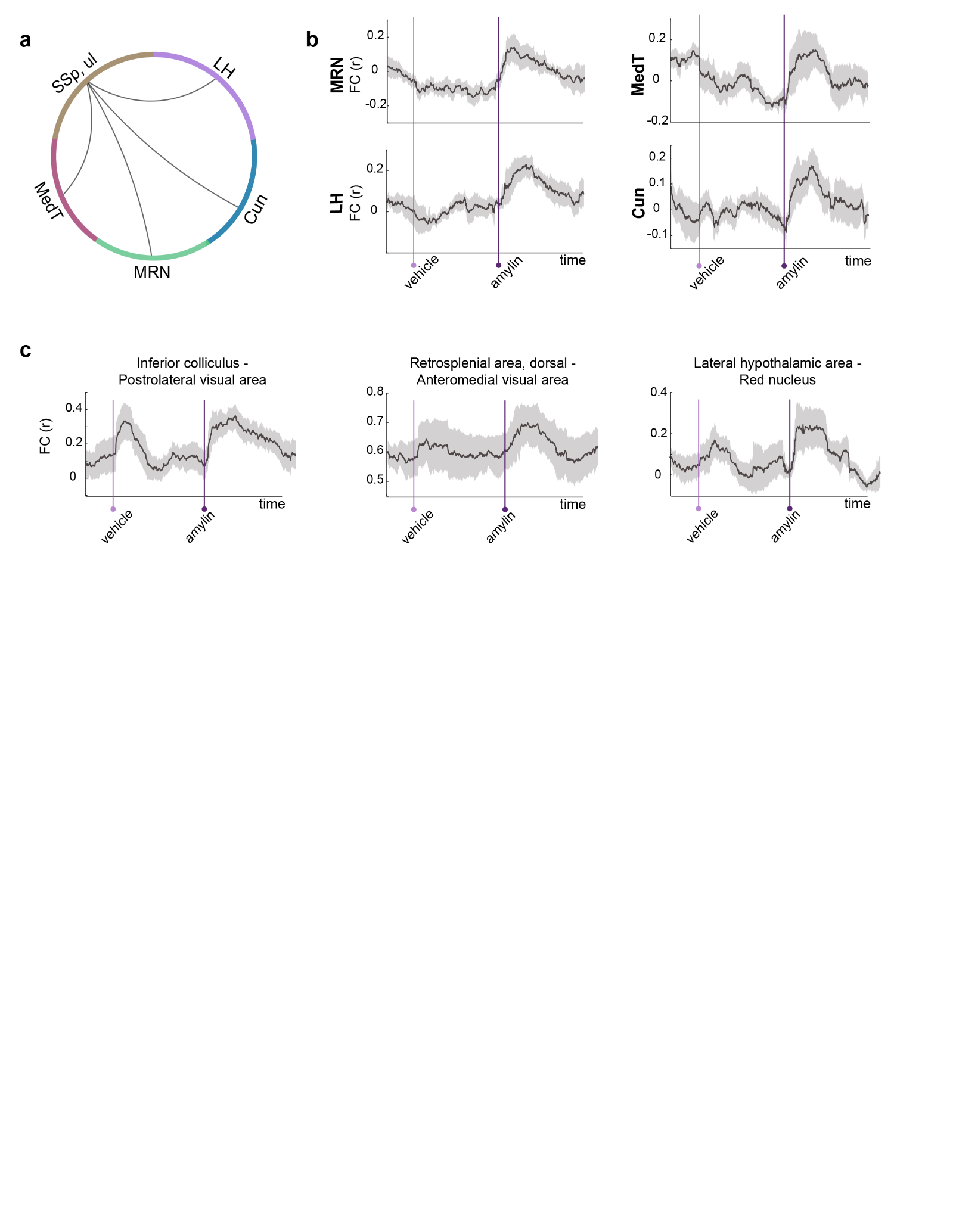


**Supplementary Figure S6.** Functional connectivity (FC) alterations in RAMP1/3 knockout (KO) mice. **a** Region pairs showing significant FC increases after amylin administration. **b** Dynamic FC trajectories for the connections in a. **c** Representative dynamic FC traces for connections that respond similarly to vehicle and amylin; these are interpreted as injection-related and not genuine amylin-driven modulations. SSp, ul, Primary somatosensory area, upper limb; LH, Lateral hypothalamic area; Cun, Cuneiform nucleus; MRN, Median raphe nucleus; MedT, Medial group of the dorsal thalamus.


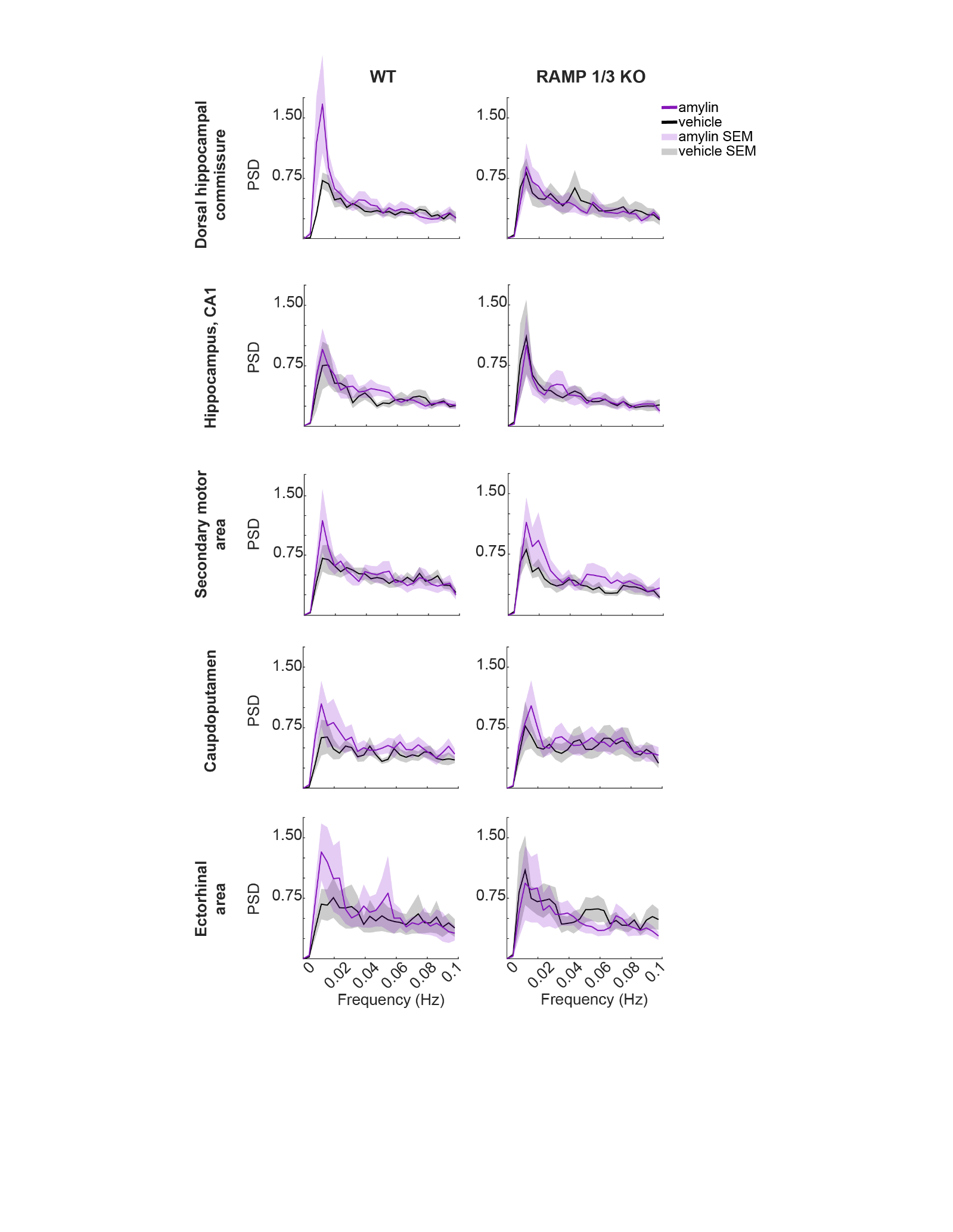


**Supplementary Figure S7.** Power spectral density (PSD) analyses of the network component time-courses in WT (left) and RAMP1/3 KO (right) mice, as depicted in Fig. 3a for amylin and vehicle conditions.

**Supplementary Table 1.** List of anatomically defined regions based on the Allen Mouse Brain Atlas used for functional connectivity analysis in this study.

| 'Primary motor area' | 'Entorhinal area, lateral part' |
| --- | --- |
| 'Secondary motor area' | 'Entorhinal area, medial part' |
| 'Primary somatosensory area, nose' | 'Subiculum' |
| 'Primary somatosensory area, barrel field' | 'Endopiriform nucleus' |
| 'Primary somatosensory area, lower limb' | 'Lateral amygdalar nucleus' |
| 'Primary somatosensory area, mouth' | 'Striatum' |
| 'Primary somatosensory area, upper limb' | 'Caudoputamen' |
| 'Primary somatosensory area, trunk' | 'Nucleus accumbens' |
| 'Supplemental somatosensory area' | 'Lateral septal nucleus' |
| 'Gustatory areas' | 'Central amygdalar nucleus' |
| 'Visceral area' | 'Globus pallidus' |
| 'Dorsal auditory area' | 'Bed nuclei of the stria terminalis' |
| 'Primary auditory area' | 'Thalamus' |
| 'Posterior auditory area' | 'Ventral posterior complex of the thalamus' |
| 'Ventral auditory area' | 'Lateral group of the dorsal thalamus' |
| 'Anterolateral visual area' | 'Anterior group of the dorsal thalamus' |
| 'Anteromedial visual area' | 'Medial group of the dorsal thalamus' |
| 'Lateral visual area' | 'Paraventricular thalamus' |
| 'Primary visual area' | 'Hypothalamus' |
| 'Posterolateral visual area' | 'Periventricular zone of hypothalamus' |
| 'posteromedial visual area' | 'Anterior hypothalamic area' |
| 'Postrhinal area' | 'Ventromedial hypothalamic nucleus' |
| 'Anterior cingulate area, dorsal part' | 'Posterior hypothalamic nucleus' |
| 'Anterior cingulate area, ventral part' | 'Lateral hypothalamic area' |
| 'Prelimbic area' | 'Arcuate hypothalamic nucleus' |
| 'Infralimbic area' | 'Zona incerta' |
| 'Agranular insular area, dorsal part' | 'Midbrain' |
| 'Agranular insular area, posterior part' | 'Superior colliculus, sensory related' |
| 'Agranular insular area, ventral part' | 'Inferior colliculus' |
| 'Retrosplenial area, lateral agranular part' | 'Substantia nigra, reticular part' |
| 'Retrosplenial area, dorsal part' | 'Ventral tegmental area' |
| 'Retrosplenial area, ventral part' | 'Midbrain reticular nucleus' |
| 'Anterior area' | 'Anterior pretectal nucleus' |
| 'Rostrolateral visual area' | 'Cuneiform nucleus' |
| 'Temporal association areas' | 'Red nucleus' |
| 'Perirhinal area' | 'Pedunculopontine nucleus' |
| 'Ectorhinal area' | 'Pons' |
| 'Anterior olfactory nucleus' | 'Nucleus of the lateral lemniscus' |
| 'Piriform area' | 'Principal sensory nucleus of the trigeminal' |
| 'Cortical amygdalar area' | 'Parabrachial nucleus' |
| 'Hippocampal formation' | 'Pontine reticular nucleus' |
| 'Field CA1' | 'Superior central nucleus raphe' |
| 'Field CA2' | 'Area postrema' |
| 'Field CA3' | 'Nucleus of the solitary track' |
| 'Dentate gyrus' |  |

**Supplementary Table 2.** Summary of amylin-induced functional connectivity changes in WT mice absent in RAMP1/3 knockout animals.

**Change**: Direction of FC modulation. ‘++’ indicates increased FC, ‘--’ indicates decreased FC.
**Duration**: Approximate duration of the FC change following amylin injection.
**Onset**: Temporal profile of the response. ‘+++’ denotes rapid-onset changes; ‘+’ indicates slower-onset responses.

| Region 1 | Region 2 | Change | Duration | Onset |
| --- | --- | --- | --- | --- |
| Area postrema | Parabrachial nucleus | -- | 10 min | +++ |
| Parabrachial nucleus | Arcuate hypothalamic nucleus | ++ | 10 min | ++ |
| Parabrachial nucleus | Paraventricular nucleus of the thalamus | ++ | 10 min | +++ |
| Parabrachial nucleus | Peduncopontine nucleus | ++ | 15 min | +++ |
| Parabrachial nucleus | Ventral posterior complex of the thalamus | ++ | 10 min | ++ |
| Parabrachial nucleus | Globus pallidus | ++ | 10 min | ++ |
| Nucleus of the lateral lemniscus | Paraventricular hypothalamic nucleus | ++ | 15 min | ++ |
| Nucleus of the lateral lemniscus | Anterior group of the dorsal thalamus | ++ | 15 min | +++ |
| Nucleus of the lateral lemniscus | Pontine reticular nucleus | ++ | 15 min | +++ |
| Nucleus of the lateral lemniscus | Arcuate hypothalamic nucleus | ++ | 10 min | ++ |
| Arcuate hypothalamic nucleus | Peduncopontine nucleus | ++ | 10 min | ++ |
| Arcuate hypothalamic nucleus | Prelimbic area | -- | 20 min | ++ |
| Lateral hypothalamic area | Central amygdalar nucleus | ++ | 10 min | +++ |
| Lateral hypothalamic area | Visceral area | ++ | 10 min | ++ |
| Lateral hypothalamic area | Inferior colliculus | ++ | 15 min | ++ |
| Inferior colliculus | Ventromedial hypothalamic nucleus | ++ | 15 min | ++ |
| Inferior colliculus | Visceral area | ++ | 10 min | ++ |
| Inferior colliculus | Nucleus accumbens | ++ | 10 min | ++ |
| Inferior colliculus | Agranular insular area, posterior part | ++ | 10 min | ++ |
| Agranular insular area, posterior part | Lateral hypothalamic area | ++ | 10 min | +++ |
| Agranular insular area, posterior part | Anterior group of the dorsal thalamus | ++ | 15 min | +++ |
| Lateral group of dorsal thalamus | Globus pallidus | ++ | 10 min | ++ |
| Lateral group of dorsal thalamus | Lateral septal nucleus | ++ | 15 min | ++ |
| Ventral tegmental area | Ventromedial hypothalamic nucleus | -- | 20 min | + |
